# Clinical Implications of Power Toothbrushing on Fluoride Delivery: Effects on Biofilm Plaque Metabolism and Physiology

**DOI:** 10.1155/2010/651869

**Published:** 2010-04-15

**Authors:** M. Aspiras, P. Stoodley, L. Nistico, M. Longwell, M. de Jager

**Affiliations:** ^1^Clinical and Scientific Affairs, Philips Oral Healthcare, 35301 SE Center Street, Snoqualmie, WA 98065, USA; ^2^National Centre for Advanced Tribology at Southampton (nCATS), School of Engineering Sciences, University of Southampton, Highfield, Southampton, SO17 1BJ, Uk; ^3^Center for Genomic Sciences, Allegheny-Singer Research Institute, 11th Floor South Tower, 320 East North Avenue, Pittsburgh, PA 15212-4772, USA

## Abstract

Dental biofilms are implicated in the formation of caries and periodontal disease. A major constituent of the supragingival biofilm is *Streptococcus mutans*, which produces lactic acid from sucrose fermentation, enhancing enamel demineralization and eventual caries development. Caries prevention through F inhibits enamel demineralization and promotes remineralization. Fluoride also exerts effects on metabolic activities in the supragingival biofilm such as aerobic respiration, acid fermentation and dentrification. In experimental *S. mutans* biofilms, adding 1000 ppm F to an acidogenic biofilm resulting from 10% sucrose addition increased pH to pre-sucrose levels, suggesting inhibition of acid fermentation. F effects on metabolic activity and sucrose utilization in interproximal plaque biofilms were also recorded. Addition of 10% sucrose reduced pH from neutral to 4.2, but subsequent addition of 1000 ppm F increased pH by 1 unit, inhibiting acid fermentation. 10% Sucrose addition also stimulated denitrification, increasing production of nitrous oxide (N_2_O). Addition of 1000 ppm F suppressed denitrification, indicating an additional mechanism by which F exerts effects in the active interproximal biofilm. Finally, fluid dynamic activity by power tooth brushing enhanced F delivery and retention in an experimental *S. mutans* biofilm, suggesting a potential novel benefit for this intervention beyond mechanical plaque removal.

## 1. Introduction

The accumulation of dental plaque biofilms plays an important role in the development of caries, gingivitis, and periodontitis. Bacteria in dental biofilms constitute a viable community of microorganisms with complex ecological relationships that influence the microenvironment in which they reside [1, 2]. As these bacteria proliferate, they utilize nutrients from their immediate environment. In the case of supragingival plaque bacteria, saliva or external dietary carbohydrates from ingested food are major nutritional sources, while for subgingival plaque bacteria, proteins from gingival crevicular fluid or tissue breakdown products are the main nutritional reservoirs [1]. The expanding biofilm forms an irregular heterogeneous sponge-like structure containing clusters of bacterial cells surrounded by channels through which liquids such as saliva, ingested fluids, or mouthwash can flow [3]. *Streptococcus mutans*, which produces lactic acid from the fermentation of sucrose and is instrumental in caries formation, is a major constituent of supragingival biofilms [4]. The cariogenic aspect of *S. mutans* biofilms is due in part to an increase in the dissolution rate of hydroxyapatite, a mineral which constitutes more than 95% of tooth enamel. As acidity increases such that the pH drops below 5, increased demineralization of the enamel surface in turn accelerates the development of cavities. In addition, cariogenic bacteria on the periphery of the cell clusters, many of which are aerobes and facultative anaerobes, actively consume dissolved oxygen, resulting in oxygen deprived niches that favor proliferation of anaerobic pathogens [5]. The progressive development of the biofilm ecosystem in terms of these aerobic or anaerobic niches is heavily influenced by acid production from fermentation of dietary sucrose and other sugars. Thus, the link that occurs between an initially cariogenic biofilm to one where anaerobic periodontal bacteria eventually predominate underscores the need for early anticariogenic interventions as evidenced by the use of fluoride. Since the topical application of fluoride rather than systemic delivery is considered most effective in promoting caries reduction, understanding the mode in which fluoride is delivered to the plaque biofilm and underlying enamel is important [6, 7]. Much of the emphasis of this article will therefore focus on the various mechanisms of fluoride interference on localized areas of biofilm physiology. In addition, the role that power toothbrushes can play in enhancing fluoride delivery into biofilms provides an expanded dimension for preexisting oral care devices to new applications, underscoring the need to continue searching for new directions in anticaries interventions.

## 2. Clinical Relevance of Fluoride as an Anticaries Intervention

The use of fluoride as a preventive measure against dental caries is well established. Comprehensive summaries on the importance of fluoride in combating caries have been published by Wefel [8], ten Cate and Featherstone [9], and Stoodley et al. [5]. The clear therapeutic effect of fluoride on dental caries was first shown in 1945 in Grand Rapids, MI, with the addition of fluoride to drinking water [10–12]. 11 years after water fluoridation in this study, 30,000 schoolchildren were monitored and found to have reduced caries incidence in excess of 60%. In subsequent years, the potential for fluorosis to develop in individuals when fluoride was added to water with already elevated natural concentrations of the mineral led researchers to determine the optimal concentration of fluoride in treated water. The concentration that would still exert an anticaries effect while minimizing the probability of developing fluorosis was determined to be approximately 1 mg/L [13]. 

Three main mechanisms have been proposed to explain the anticaries effect of fluoride. Firstly, fluoride enhances the resistance of enamel to increased acid attack by reducing enamel calcium hydroxyapatite solubility in acid. This occurs through the replacement of ions in calcium hydroxyapatite to form fluorapatite, reducing the susceptibility to acid 10-fold by lowering the onset of acidic dissolution from pH 5.5 to approximately pH 4.5 [8, 14]. Secondly, fluoride has been linked to reduced acid production by mutans streptococci and lactobacilli [15]. This can occur through inhibition of the metabolic and physiological pathways in the cariogenic biofilm that result in lactic acid production [15, 16]. Thirdly, fluoride can inhibit demineralization (enamel dissolution) and enhance remineralization (enamel deposition) in tooth enamel, positively impacting the ongoing process of remineralization-demineralization in tooth enamel [17]. If exposure to acid is short, saliva will raise the pH naturally so that the enamel loss can be repaired through remineralization. However, continued exposure to acid (e.g., continuous sucking on sugar-containing candies or sipping sugary drinks) can create a situation whereby the remineralization rate may be insufficient to repair the loss from demineralization, increasing the likelihood of caries development [18]. Hence, the right balance in the rates of demineralization and remineralization influences the success of caries reduction following implementation of a caries protection strategy [19–21].

## 3. Fluoride Effects on Aerobic Respiration, Acid Fermentation and Nitrification

As mentioned, fluoride can affect metabolic conditions in the cariogenic biofilm. Microsensors such as microelectrode probes can be used to evaluate local physiological conditions in the cariogenic biofilm, particularly the *S. mutans* biofilm established through in vitro models. Microelectrodes have tip diameters of less than 10 *μ*m, which is on a spatial scale relevant to plaque biofilms which are usually less than 1 mm thick and composed of cell clusters with diameters on the order of tens to hundreds of microns. Some of these localized conditions include oxygen (oxygen probe for measuring aerobic respiration and nonrespiratory oxygen consumption), pH level (pH probe for measuring acid fermentation), and oxides of nitrogen (nitrous oxide (N_2_O) and nitrate (NO_3_) probe for measuring denitrification, a form of anaerobic respiration), before and following sucrose consumption. In addition, the effect of fluoride in the context of these physiological processes in the biofilm can be assessed by evaluating fluoride ion transport and retention through the use of specialized diffusion chambers [18]. 

**Figure 1:**
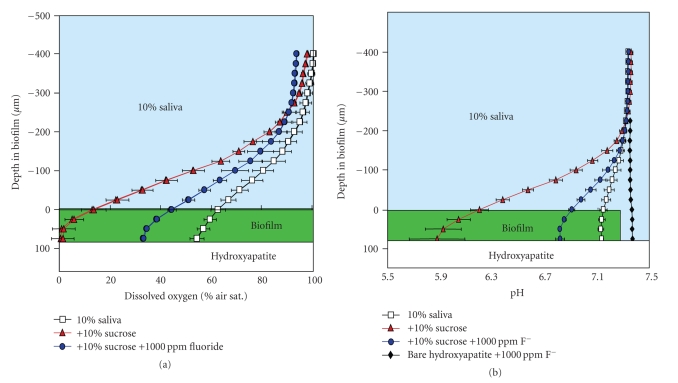
(a) Dissolved oxygen profile in 10% saliva after 10% sucrose addition increases anaerobicity to 50 *μ*m. F addition reduced biofilm activity so that dissolved oxygen increased to approximately 35% of air saturation. (b) Adding 10% sucrose decreased pH from 7.1 to 5.9 due to sucrose fermentation. F addition increased pH back to 6.8, suggesting inhibition of fermentation.

**Figure 2:**
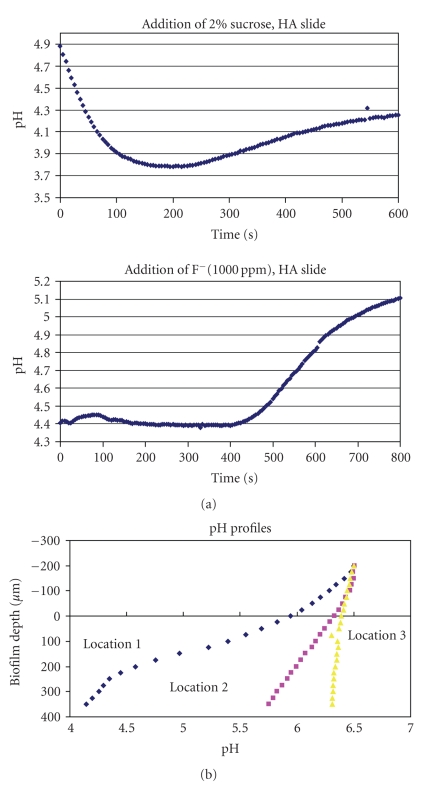
(a) *S. mutans* biofilm is grown on hydroxyapatite slides with microelectrode measurements revealing a drop in pH within the first 4 minutes after addition of 2 % sucrose. Removing sucrose and adding F raises pH after 6-7 minutes of intervention (graph redrawn from [5]). (b) Variability of microelectrode profiles within the biofilm after sucrose addition. In location 1 pH dropped to 4.2, while little change was seen in location 3.

**Figure 3:**
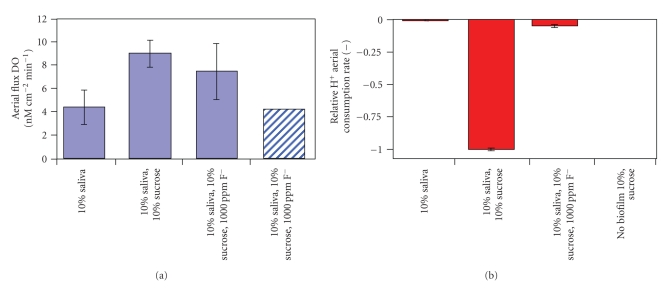
(a) In an *S. mutans* biofilm, addition of sucrose increased respiration rate by over 100% while fluoride addition reduced aerobic respiration rate by 17%. The aerobic respiration rate is shown in ex vivo interproximal plaque for comparison (hatched bar). (b) Negative H^+^ consumption indicates acid production. The addition of sucrose increased activity by a factor of 170 while the addition of F significantly inhibited acid fermentation. Data for a sterile system are shown as a negative control.

Fluoride effects on biofilm physiology include influencing the localized anaerobic and acidic microenvironments found near the surface of the biofilm, promoting acid-loving bacteria that play a role in cariogenic biofilms [18]. Previous work by Stoodley et al. [5] used oxygen electrodes to measure the effect of adding 1000 ppm fluoride on dissolved oxygen levels in an *S. mutans* biofilm grown on hydroxyapatite coated slides (to simulate tooth surface enamel) in a drip flow reactor and then placed in a flow cell system. Fluoride increased the level of dissolved oxygen to 35% of air saturation in the biofilm, thereby creating a less favorable environment for pathogenic anaerobes. (Figure 1(a)) The same study also used pH probes to measure the effect of adding fluoride on biofilm pH profiles. Addition of 10% sucrose dropped pH from 7.1 to 5.9, while addition of F increased pH back to 6.8, showing that fluoride inhibits acid fermentation by increasing pH following the low pH levels generated by sucrose-driven fermentation (Figure 1(b)). The temporal element of acid fermentation and effect of subsequent fluoride addition was also explored in another experiment as shown in Figure 2(a). Acid production following addition of 2% sucrose resulted in a continuous drop in pH within the first 4 minutes. Removal of sucrose and addition of fluoride raised the pH after 6-7 minutes of intervention, reflecting the progressive dispersion of fluoride within the biofilm. pH variability also occurs at different sites within the *S. mutans* biofilm grown on hydroxyapatite slides. As Figure 2(b) demonstrates, placement of pH microelectrodes at three different sites within the biofilm after sucrose addition revealed substantial variability in pH response at different depths within these three microenvironments. Sucrose consumption and subsequent acid respiration resulted in a pH decline from approximately 7 to as low as 4.2, which is sufficient to cause tooth surface enamel erosion. Finally, a related experiment exploring the effects of fluoride used aerial flux to measure aerobic respiration and acid fermentation activity in a 75 *μ*m thick *S. mutans* biofilm. Results of this experiment are shown in Figures 3(a) and 3(b). Effect of 10% sucrose addition on aerial O_2_ flux increased the aerobic respiration rate by over 100% while subsequent fluoride addition (1000 ppm) reduced aerobic respiration rate by 17%. Relative proton (H^+^) aerial consumption showed increased negative activity (i.e., H^+^ production resulting in acidification) by a factor of 170 following sucrose addition, with subsequent addition of fluoride significantly inhibiting acid fermentation. In summary, what these various experiments validate is the role of fluoride in mitigating an acidic environment in the dental plaque biofilm by inhibiting acid fermentation of resident cariogenic bacteria such as *S. mutans*. Reduction of the acidic environment indirectly favors proliferation of the beneficial nonmutans streptococci that are harmed by the presence of high acid levels. The clinical significance of these health associated bacteria in the biofilm is that their presence is generally indicative of good oral health and hygiene.

These findings might be generalizable to other areas of the mouth. Experiments by Stoodley et al. [22] on tonsillolith biofilms have shown evidence of metabolic stratification similar to that found in dental biofilms. For instance, an overlapping denitrification zone was found between an upper layer characterized mainly by aerobic respiration and a lower (deeper) layer where acid fermentation predominated. Denitrification is the result of the decomposition of nitrite into nitrous oxide (N_2_O) and sometimes nitric oxide (NO). Phagocytic cells, which form part of the innate host defense system, produce nitric oxide (NO) (which is a strong oxidizer) from arginine, specifically to attack bacteria as part of the phagocytic oxidative burst. However, bacteria can also produce nitric oxide (NO) from the reduction of nitrate ion (NO_3_^−^). Some bacterial species have evolved reductases that reduce nitric oxide (NO) further to nitrous oxide (N_2_O) or nitrogen (N_2_), rendering it harmless. It is not clear whether the ability of bacteria to degrade nitric oxide (NO) was an evolutionary adaptation to counter the bactericidal activity of phagocytic nitric oxide (NO) or to detoxify nitric oxide (NO) as a waste product of denitrification. Another possibility is that in an acid environment nitrite spontaneously oxidizes to nitric oxide (NO) so that the bacterial reduction of nitrate (NO_3_^−^) to nitrite (NO_2_^−^) in such an environment might be utilized by bacteria, not primarily for energy, but to attack competitive bacteria. Such a strategy would require that these bacteria have defenses against their own arsenal. A major contribution of this study was the use of microelectrodes to measure chemical gradients in biofilms grown on a mucosal surface. In addition, the use of tonsilloliths as a model for dental biofilms allowed for cross functional insights into similarly stratified physiological activities present in dental biofilms, notably interproximal plaque.

**Figure 4:**
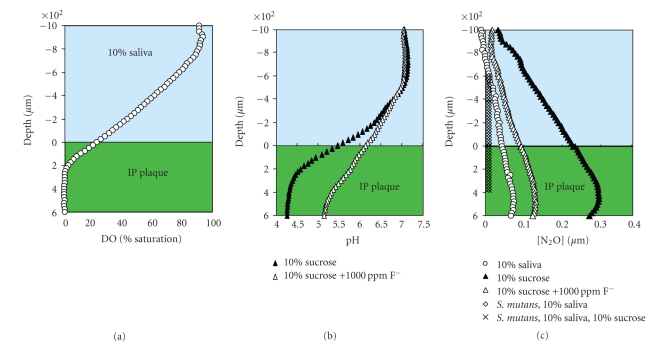
Metabolic activity in interproximal plaque. (a) In 10% saliva the plaque was anaerobic below a depth of 200 *μ*m. (b) The addition of 10% sucrose reduced the pH from neutral to 4.2 within the dental plaque biofilm. NaF increased the pH by approximately 1 unit suggesting that acid fermentation had been inhibited. (c) N_2_O production in the biofilm demonstrated that the interproximal plaque was denitrifying. Addition of sucrose stimulated denitrification while F suppressed it.

To observe if select in vitro physiological activities observed earlier in *S. mutans* biofilms extended to ‘‘natural” multispecies dental biofilms, the activities were examined in interproximal plaque. Experiments were conducted on ex vivo interproximal plaque immersed in 10% saliva. Oxygen, pH, and nitrous oxide (N_2_O) electrodes were used to measure aerobic respiration, acid fermentation, and denitrification, respectively, after sequential additions of sucrose and fluoride, or nitrous oxide (N_2_O), sucrose, and fluoride for the denitrification studies (Figures 4(a), 4(b), and 4(c)). In the presence of 10% saliva alone, aerobic respiration predominates in ex vivo plaque where anaerobicity occurs below 200 *μ*m. Supplementation with 10% sucrose induced acid fermentation, reducing pH from approximately 7 to 4.2. Subsequent addition of 1000 ppm fluoride increased pH to approximately 5.2, suggesting fluoride inhibition of acid fermentation. Nitrous oxide (N_2_O) production after addition of sucrose provided evidence for denitrification in the interproximal biofilm. Following addition of fluoride, denitrification was suppressed, revealing yet another pathway of fluoride-induced inhibition in the active dental biofilm.

## 4. Dynamics of Fluoride Transport and Retention in *S. mutans* Flowcell Biofilm

As previously stated, the effectiveness of the topical application of fluoride in reducing caries underscores the need to understand how fluoride can be delivered to the plaque biofilm and ultimately to underlying enamel. Repeated exposure of plaque to fluoridated drinking water or dentifrice enables fluoride to bind to the sticky polysaccharide slime in the biofilm [23]. Even when the fluoride source is no longer present, bound fluoride in the plaque biofilm is slowly released over time, which can prolong anticaries activity. The biofilm acts as a storage reservoir for fluoride (and other ions such as calcium and phosphate) causing enhanced fluoride retention and exchange between these ions and tooth enamel, increasing the length of remineralization time to combat caries [18]. However, there is still insufficient knowledge on the exact mechanisms by which biofilms actively control fluoride passage through their complex layers, other than passive diffusion of fluoride through inert areas of the biofilm where there is virtually no fluid flow. Transport of small molecules or ions such as fluoride by diffusion is relatively fast across minute distances, but the time to attain a certain concentration at the base of the biofilm increases with the square of the thickness of the biofilm. Biofilm cell aggregates impede fluid flow (and hence fluoride mobility) through the cell clusters and to the tooth enamel surface itself, the ultimate target of fluoride activity.

## 5. Role of Toothbrushing in Fluoride Delivery

Even though fluoride was shown to be transported and retained in the *S. mutans* biofilm on account of diffusive flow alone, the role of fluid dynamic activity generated by power toothbrushes in enhancing fluoride delivery has not been sufficiently explored. Power brushing is designed to mechanically remove as much plaque as possible, particularly in inaccessible areas of the oral cavity. Such areas include fissures, interproximal and even subgingival areas, and possibly less exposed locations of the dentition such as posterior teeth. Increased penetration of fluoride into the biofilm through hydrodynamic forces could also enhance the period of fluoride retention and prolong its efficacy. Since topical rather than systemic fluoride delivery results in caries protection, the efficacy of fluoride delivery to problematic sites is as important as concentration of salivary fluoride and frequency of fluoride exposure. Dental plaque biofilms formed in stagnant areas within the dentition can still result in caries if not physically removed or chemically managed. The motion from a sonic toothbrush has been demonstrated in vitro to drive fluid dynamic forces beyond the reach of the bristles into inaccessible interproximal spaces, resulting in biofilm removal in these areas [24]. As a result, it is conceivable that fluid dynamics can also assist in the penetration of fluoride deeper into those areas of the interproximal biofilm that remains post brushing, allowing delivery of the extra few parts per million (ppm) of fluoride that is considered beneficial for added protection against caries. 

A previously published experiment by Stoodley et al. [18] demonstrated fluoride delivery and retention into an in vitro* S. mutans* biofilm using a dual chamber system. Both chambers were separated by a permeable membrane colonized with *S. mutans* biofilm representing dental plaque to simulate in vivo interproximal plaque biofilm. The objective was to measure how quickly sodium fluoride passed through the colonized membrane from one chamber into the other during sonic brushing [18, 25–27]. A primary chamber served as the brushing chamber while a secondary measurement chamber served as the fluoride detection chamber to measure accumulating fluoride. A fluoride electrode in the measurement chamber measured how much fluoride was driven into it through the biofilm membrane following powered brushing in the primary chamber. The brushing chamber was filled with 1100 ppm fluoride solution and over a 4-minute monitoring period, the concentration in the brushing chamber never fell to less than 1050 ppm, suggesting that the concentration gradient driving the fluoride flux would remain more or less constant. Even with no brushing, fluoride concentration increased from 0.4 ppm to 0.5 ppm after 4 minutes due to the difference in fluoride concentration between the two chambers (passive diffusion). But with active brushing, the delivery of fluoride through the biofilm membrane increased considerably over a 4 minute brushing period for two power toothbrushes, with fluoride concentration measured in the measurement chamber at 0.65 ppm for one brush and at 0.8 ppm for the other, sonic brush. Fluoride delivery rate through the colonized membrane was measured as the mass transfer rate coefficient, which was significantly greater with power brushing than with passive diffusion alone.

## 6. Conclusions

The complexity of fluoride effects on microbial physiology can be summarized into three factors: saliva and overlying fluids, the plaque biofilm, and the underlying tooth enamel, into which fluoride exerts its main clinical benefits. In addition to the ionic interactions that occur among calcium, phosphate, and fluoride, other factors include transient pH that can range from pH 4 to 7, localized aerobic/anaerobic niches, temporal effects (seconds to minutes), and distance scales (micrometers to millimeters). The success of measurements to capture these physiological parameters depends on employing tools such as microelectrodes and compelling in vitro systems to model real life dental plaque biofilms.

The use of microelectrodes has proven to be a promising tool in studying localized fluctuations within in vitro plaque biofilms of physiologically relevant parameters such as pH, O_2_, and nitrous oxide (N_2_O) and has the possibility of being utilized extraneous beneficial agents such as fluoride. It is expected that further exploration into fluoride effects on biofilms in other niche sites such as the ‘‘subgingival” sites of the typodont would enhance previous observations that were recorded for the similarly less accessible interproximal sites.

Finally, increasing evidence suggesting the link between power toothbrushes and enhanced fluoride effects on the dental biofilm serves to steer new avenues of exploration for clinical benefits of power tooth brushes extending beyond mechanical bristle activity and plaque removal. The potential for enhanced fluoride delivery and retention into plaque biofilms through indirect but potent fluid dynamic action becomes even more useful where biofilms are located in hard to access areas, thus benefiting underlying enamel underneath these biofilms. Even the nature of brushing can impact efficacy of fluoride delivery. A four-day clinical trial revealed that sonic brushing increased the concentration of retained fluoride in plaque biofilm increased by greater than 40% compared to rotary brushing, manual brushing, and manual brushing and flossing [28]. Further research into the physical relationships among power brushing, fluid dynamic activity, and the role of localized oxygen gradients in oral biofilms should be explored in the context of increasing fluoride retention and delivery. Many of the more pathogenic, anaerobic bacteria reside deeper in the plaque biofilm where the availability of oxygen is low and where they are protected from chemotherapeutic agents. However, this environment also represents a target area for potentially successful interventions by increasing oxygen availability and by delivering antimicrobials directly to these anaerobes via power brushing. Meanwhile, the opportunity for delivering and retaining other broad-based anticariogenic or antimicrobial agents into dental plaque biofilms should be considered in developing novel innovative approaches to caries management, whether as an ancillary benefit of power brushing or the main benefit of an intervention where directed delivery of the anticaries agent is the primary objective.
